# Recurrence Incidence in Differentiated Thyroid Cancers and the Importance of Diagnostic Iodine-131 Scintigraphy in Clinical Follow-up

**DOI:** 10.4274/mirt.35220

**Published:** 2016-06-06

**Authors:** Filiz Hatipoğlu, İnanç Karapolat, Özgür Ömür, Ayşegül Akgün, Ahmet Yanarateş, Kamil Kumanlıoğlu

**Affiliations:** 1 Sifa University Faculty of Medicine, Department of Nuclear Medicine, Izmir, Turkey; 2 Ege University Faculty of Medicine, Department of Nuclear Medicine, İzmir, Turkey; 3 İzmir Dr. Suat Seren State Hospital, Clinic of Nuclear Medicine, İzmir, Turkey

**Keywords:** differentiated thyroid cancer, recurrence, iodine-131 scintigraphy

## Abstract

**Objective:**

Differentiated thyroid cancers (DTC) are tumors with good prognosis. However, local recurrence or distant metastasis can be observed. In our study, we aimed to investigate the incidence of recurrence and the importance of diagnostic iodine-131 whole body scan (WBS) in clinical follow-up in patients with DTC.

**Methods:**

The clinical data of 217 patients with DTC who were followed-up more than 3 years were reviewed retrospectively. The incidence of recurrence was investigated in a group of patients who had radioactive iodine (RAI) treatment and showed no sign of residual thyroid tissue or metastasis with diagnostic WBS that was performed at 6-12 months after therapy and had a thyroglobulin (Tg) level lower than 2 ng/dl.

**Results:**

At the time of diagnosis, ten cases had thyroid capsule invasion, 25 cases had extra-thyroid soft tissue invasion, 11 patients showed lymph node metastasis and four patients had distant organ metastasis. One hundred forty-five patients had RAI treatment at ablation dose (75-100 mCi), whereas 35 patients had RAI treatment at metastasis dose (150-200 mCi). Thirty-seven patients with papillary microcarcinoma did not receive RAI treatment. In 12 (%7.5) of the 160 patients who were considered as “successful ablation”, a recurrence was identified. Recurrence was detected by diagnostic WBS in all cases and stimulated Tg level was <2 ng/dL with the exception of the two cases who had distant metastasis.

**Conclusion:**

Identification of pathological findings with WBS in patients who developed local recurrence in the absence of elevated Tg highlights the importance of diagnostic WBS in clinical follow-up.

## Abstract (tr)

**Amaç:**

Diferansiye tiroit karsinomları (DTK) iyi prognoza sahip tümörlerdir. Ancak lokal rekürrens ve uzak metastaz izlenebilmektedir. Çalışmamızda DTK’lı olgularda rekürrens sıklığının ve tanısal iyot-131 (I-131) tüm vücut tarama sintigrafisinin klinik izlemdeki öneminin araştırılması amaçlandı.

**Yöntem:**

İzlem süresi 3 yılın üzerinde olan iki yüz on yedi DTK’lı olgunun klinik verileri retrospektif olarak incelendi. Radyoiyot (RAI) tedavisi alan hastalardan tedavi sonrası 6. ay-1. yıl tanısal I-131 tarama sintigrafisinde rezidüel tiroit dokusu ve metastaza ait bulgu saptanmayan, tiroglobulin (Tg) düzeyi 2 ng/dl’nin altında olan grupta rekürrens sıklığı araştırıldı.

**Bulgular:**

Tanı anında on olguda tiroit kapsülü, 25 olguda ekstratiroidal yumuşak doku invazyonu, 11 hastada lenf nodu metastazı ve dört hastada uzak metastaz mevcuttu. Yüz kırk beş olgu ablasyon dozunda (75-100 mCi), 35 olgu metastaz dozunda (150-200 mCi) RAI tedavisi almıştı. Papiller mikrokarsinom tanılı 37 hasta ise RAI tedavisi almamıştı. Tedavi başarısına karar verilen 160 hastanın 12’sinde rekürren hastalık saptandı (%7,5). Olguların tümünde rekürrens I-131 tarama sintigrafisi ile saptanmış olup uzak metastazı olan iki hasta dışında stimüle Tg değerleri <2 ng/dL idi.

**Sonuç:**

Lokal rekürrens gelişen tüm olgularda stimüle Tg yüksekliği olmaksızın I-131 tarama sintigrafisi ile patolojik bulguların saptanması klinik izlemde tanısal I-131 taramanın önemini vurgulamaktadır.

## INTRODUCTION

Differentiated thyroid cancers (DTC) is a group of tumors with slow growth potential (1); long survival and a good clinical outcome is common, even in a patient with metastasis, when they are adequately treated with effective therapeutic approaches such as surgery, radioactive iodine (RAI) and thyroid hormone suppression (2). The 10-year survival rate in these tumors with good prognosis is above 90%. Nevertheless, local-regional recurrence or distant metastasis, especially in the first year of diagnosis, can be observed in 20% and 5-10% of the cases, respectively (3). Even though indicators of treatable recurrences and detrimental progress usually manifest within the first 5-10 years, life-long follow-up is critical since late mortality and recurrence is not uncommon (4). Traditionally, patients are followed-up according to prognostic factors, serum thyroglobulin (Tg) level and ultrasound (US) findings, anti-Tg antibody (anti-T) measurements along with concurrent whole body scintigraphy with I-131 (I-131 WBS) following thyroid stimulating hormone (TSH) stimulation at 6-12 months after surgery and ablation (5). The cervical US, serum Tg, and I-131 WBS are powerful tools to detect recurrence. Additional information can be achieved by computed tomography (CT) and magnetic resonance imaging (MRI) (6). Although I-131 scanning has a very high specificity of 99-100%, the rate of I-131-positive recurrences is about 50-60% in papillary and 64-67% in follicular thyroid cancer. In patients with I-131 negative recurrence, in addition to conventional radiologic imaging modalities, F-18 fluorodeoxyglucose (FDG) positron emission tomography (PET) and somatostatin receptor scintigraphy may yield significant information in detecting recurrence and metastasis (7). The aim of this study was to calculate the incidence of recurrence and explore which diagnostic tool was more reliable in detecting recurrence in patients with DTC.

## MATERIALS AND METHODS

### Patients

We retrospectively reviewed clinical data pertaining to 217 patients [35 males (16%) and 182 females (84%)] who presented to the Department of Nuclear Medicine, Faculty of Medicine, Ege University with post-surgery DTC between 1991 and 2006, and regularly attended follow-up control visits for a period of 3 to 22 years. Ablation was deemed successful in patients who received RAI therapy after surgery, did not show any sign of residual thyroid tissue or metastasis on diagnostic I-131 WBS performed 6-12 months after RAI therapy, and had serum Tg level <2 ng/dL in the stimulated period. Recurrence was defined as detection of a tumor in the thyroid bed, cervical lymph nodes or in distant organs. When recurrence was identified, diagnostic I-131 WBS, serum Tg level, and US findings were assessed and compared, while all clinical data of the patients were reviewed. A detailed information was given to all patients before the procedure to be applied and all patients signed informed consent forms. Ethics committee approval was not received due to the retrospective design of the study.

### Radioactive Iodine Treatment and Follow-Up Protocol

For RAI treatment, TSH stimulation was induced after all patients were instructed not to use thyroid hormone replacement after thyroid surgery or to stop using the medication at least 4 weeks before therapy if thyroid hormone replacement had already been started. Before RAI treatment, patients were given a low-iodine diet for at least 2 weeks and asked not to use any medication or radiographic contrast agent that would decrease radioiodine uptake. Once TSH level was above 30 μIU/mL, 75-100 mCi I-131 was given per-orally for ablation, 150-175 mCi for extra-thyroid soft tissue invasion and lymph node metastasis, and 175-200 mCi for distant metastasis. I-131 WBS was performed on day 10 after RAI treatment, and diagnostic I-131 WBS was carried out under TSH stimulation with concurrent serum Tg, anti-T and TSH measurements by immunoradiometric assay method at approximately 6, 12, 18, 60 (5 years), 120 (10 years) and 180 months (15 years). Prior to diagnostic I-131 WBS, all patients were given a low-iodine diet for 2 weeks and asked to refrain using any medication or radiographic contrast agent that would decrease radioiodine uptake. Patients stopped using L-thyroxine hormone 6 weeks before the scan and used T3 in the first 4 weeks instead. In the last 2 weeks, TSH stimulation was induced by terminating T3, rendering endogenous TSH level to exceed 30 μIU/mL. For diagnostic I-131 WBS, 5 mCi I-131 was given orally following TSH stimulation, and anterior and posterior projection images were acquired after 48 hours. The post-treatment and diagnostic I-131 scans were carried out using dual-head gamma camera (Infinia, General Electric Medical Systems) equipped with high-energy parallel-hole collimators. Patients were imaged in the supine position. A 10% energy window around the 364 keV energy peak of I-131 was used. In addition, annual US was carried out throughout the follow-up period and patients were assessed by other imaging modalities when necessary.

### Statistical Analysis

All statistical analyzes were carried out using “SPSS 13.0 for Windows” (SPSS, Inc., Chicago, IL., USA) statistical software. Descriptive statistics of the patients are presented as mean±standard deviation and/or median (minimum-maximum).

## RESULTS

**Figure 1:**
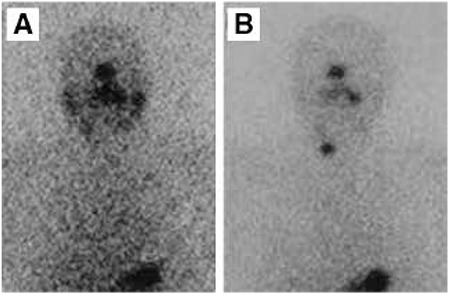
Depicted post-therapeutic and follow up I-131 whole-body scintigraphy images of a 41-year old female patient operated for papillary thyroid cancer and received 150 mCi radioactive iodine treatment. A) I-131 whole-body scintigraphy performed at 6 months post-radioactive iodine treatment showing no activity in the cervical region consistent with ablation,B) I-131 whole-body scintigraphy of the same patient performed 5 years after radioactive iodine treatment revealing increased focal activity in the right lobe of thyroid consistent with recurrent malignancy (stimulated serum thyroglobulin level of the patient during this period was 1.02 ng/dl)

**Figure 2:**
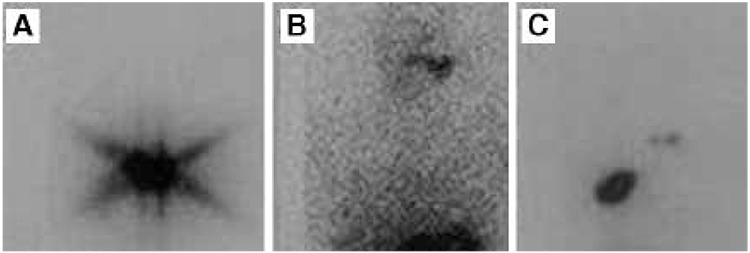
Shows post-therapeutic and follow up I-131 whole-body scintigraphy images of a 55-year old male patient operated for follicular thyroid cancer and received 100 mCi radioactive iodine treatment. A) I-131 whole-body scintigraphy performed 10 days after radioactive iodine therapy displayed high activity in the cervical region by residual tissue, B) I-131 whole-body scintigraphy of the same patient performed at 6 months after radioactive iodine therapy showed no activity consistent with ablation of residual tissue, C) I-131 whole-body scintigraphy of the same patient performed about 12 years after radioactive iodine treatment revealed increased focal activity in the thyroid bed consistent with recurrent malignancy, as well as intense uptake in the right upper thoracic region as a result of newly developed bone metastasis to the scapula (stimulated serum thyroglobulin level of the patient during this period was 187 ng/dl)

The mean age of the 217 patients, 182 females (84%) and 35 males (16%), was 52.17±13.18 years, and the mean follow-up period was 6.26±3.0 years. All patients had either total or near-total thyroidectomy. Postoperative histopathologic examination showed follicular cancer in 32 (14.7%) and papillary cancer in 185 (85.3%) cases. The mean tumor diameter was 1.84±1.47 cm. At the time of diagnosis; 10 cases had thyroid capsule invasion (4.6%), 25 had extra-thyroid soft tissue invasion (11.5%), 11 had lymph node metastasis (5.1%), and 4 had distant metastasis (1.8%). One hundred forty-five cases (66.8%) received RAI therapy at ablation dose (75-100 mCi) while 35 cases (16.1%) had RAI therapy at metastasis dose (150-200 mCi). Thirty-seven cases (17.1%) diagnosed with papillary microcarcinoma with a tumor size smaller than 1 cm did not receive RAI therapy. Treatment was considered successful in 160 cases when diagnostic I-131 WBS performed at 6-12 month post-therapy did not show abnormal I-131 uptake in the thyroid bed or any other region, and the stimulated serum Tg level was lower than 2 ng/dl. During follow-up of these patients who were considered as “successful ablation”, I-131 WBS revealed local recurrence in 10 and distant metastasis in two patients. Recurrence was detected at years 12 and 16 in patients who had distant metastasis, and within the first 5 years in the ten patients who had a local recurrence (earliest 3rd year). Of these 12 patients, nine were female (75%) and three were male (25%), with a mean age of 53.33±13.46 years and a mean tumor size of 1.62±0.83 cm at the time of diagnosis. Of these twelve patients, one patient (8.3%) was followed-up for follicular and 11 patients (91.7%) for papillary thyroid cancer. One patient had extra-thyroid soft tissue invasion, and two patients had thyroid capsule invasion. During the 6-12 months follow-up period of these patients, Anti T was negative and with the exception of the two patients who had distant metastasis the stimulated Tg was <2 ng/dl. The median value of stimulated Tg was 0.2 ng/dL (minimum 0.01 ng/dL and maximum 1.1 ng/dl). I-131 WBS identified a recurrence in all these patients (Figure 1). At year 16, stable nodules found in thoracic CT images of a patient who had high Tg level (56 ng/dL) and right-sided pathologic pulmonary uptake on I-131 WBS were not interpreted as recurrence, but F-18 FDG-PET scan performed in the same year reported metastasis to the right lung. In another patient, increased activity in the right upper thoracic region was found on I-131 WBS at year 12 as a result of newly developed bone metastasis to the scapula (Figure 2). The bone metastasis was confirmed by MRI. Both cervical US and I-131 WBS revealed residual thyroid tissue in five patients with local recurrence, otherwise cervical US did not show any significant sign of recurrence while I-131 WBS revealed residual thyroid tissue in the other five patients with local recurrence. The frequency of recurrence in DTC patients was calculated as 7.5%. The clinical data of the patients with DTC are summarized in Table 1.

## DISCUSSION

DTC comprise more than 90% of all primary thyroid cancers. The ten-year survival rate in DTC is reported as 80-95% (8); this rate is determined as 57% even in patients with pulmonary metastasis (9). However, a persistent or recurrent disease can be found in 5-24% of patients despite slow-growth and good prognosis (8). In the present study, the frequency of recurrence was calculated as 7.5%. The frequency of recurrence in low-risk DTC patients in the literature is stated as 0.5-0.6% (10). In a study, the most common type of recurrence was regional recurrence (53%), followed by local (28%), distant (13%) and combined (6%) recurrences (11). In the present study, the number of patients with local recurrence was higher than those with distant metastasis.

In tumor node metastasis classification, the presence of a positive lymph node in a DTC patient aged 45 years and above is an independent risk factor for recurrence though it has been reported that mortality rate is not affected by this parameter. Numerous researchers argued that recurrence rate was related to the number and localization of positive lymph nodes (12,13). For instance, a positive lymph node in the lateral compartment is associated with a significantly higher recurrence rate and shorter recurrence time as compared to a node in the central compartment (14). Even though lymph node metastasis was present in 5.1% of patients in our study group, none of the patients who developed recurrence had lymph node metastasis at the time of diagnosis. Due to the limited number of subjects in the research group, we were unable to compare the recurrence rates between patients with and without lymph node metastasis. Palme et al. (15) argued that male sex, advanced stage at diagnosis and extra-thyroid dissemination were independent determinants of DTC recurrence. Mazzaferri and Kloos (16) reported that the frequency of recurrence of papillary thyroid cancer was higher when patients were 20 years old or younger in comparison to 20-59-year-old patients. The effects of histologic type, tumor size, age and sex on persistent or recurrent disease in well-DTC have been investigated in a retrospective study covering the period 1979-2007. In contrast to previous studies, authors of this study argued that tumor size or sex was not a determinant of recurrence while age had a prognostic value in patients with radio-iodine treatment following total or near-total thyroidectomy (17). We were unable to establish whether a statistically significant difference existed between the patients who developed recurrence and the general group in terms of age, sex, tumor size, extra-thyroid involvement since the number of subjects was limited. However, the male/female ratio was higher in our patient group who developed recurrence.

Treatment of DTC includes total thyroidectomy, ablation with RAI, and suppression with L-thyroxine (18). Even after a successful initial treatment, the manifestation of clinical recurrence in as many as 20% of patients, and the disease-related mortality rate of 50-60% in patients with recurrence following curative treatment highlight the importance of life-long follow-up (19). I-131 WBS and serum Tg measurements with or without TSH stimulation are the most commonly used methods to follow-up DTC patients after surgery and ablation treatment. Furthermore, at follow-up visits, US of the central compartment and regional lymph nodes along the cervical chain need to be carried out periodically, at postoperative 6 and 12 months and afterward, depending on the individual risk of recurrent disease (5). Tg is a glycoprotein specific to differentiated thyroid tissue. Stimulated serum Tg level ≥2.5 ng/dL after thyroidectomy and radioiodine ablation in anti-T negative patients indicates the persistence of thyroid tissue or recurrence (20). However, a recurrent and metastatic disease cannot be detected by serum Tg measurement alone in 10% of the cases (21). Another method to detect recurrent or metastatic disease is I-131 WBS (4). Metastatic or recurrent cancers can concentrate as much as 80% iodine. I-131 WBS enables detection of recurrence before clinical signs are evident (22). In the present study, with the exception of two patients with distant metastasis, stimulated Tg values of all patients who had recurrence were lower than 2 ng/dl, and recurrence was identified by I-131 WBS during standard follow-up evaluation. However, since the negative predictive value of undetected stimulated serum Tg measurement is 100%, some authors suggested that I-131 WBS did not yield further information in addition to Tg measured under stimulation in high-risk patients while some authors did not recommend I-131 WBS in low-risk patients at all (5,23). In routine practice, I-131 WBS is recommended in anti-T positive patients (23). I-131 WBS has a high specificity rate in demonstrating recurrences though a third of the patients with recurrence lose iodine avidity (24). In such patients, US is especially preferred to assess lateral and central cervical compartments. The sonographic examination is helpful in terms of local-regional recurrence observed in as many as 20% of patients with thyroid cancer. Studying a series of patients with recurrence in the thyroid bed, Frasoldati et al. (25) reported that US was more sensitive in detecting recurrence than serum Tg level and I-131 WBS. In this study, Tg level measurement after cessation of T4 by immunoradiometric assay, I-131 WBS, and US have been carried out in an attempt to compare their results with histologic findings of fine needle aspiration biopsy, and the sensitivity of US has been calculated as 94.1%. Even though cervical US is one of the preferred methods in patients suspected of having a local recurrence, small lymph nodes may be overlooked due to post-operative granulomatous tissue, resulting in an inaccurate diagnosis. Lending support to this view, in our study, cervical US did not reveal any pathologic signs in 5 of the patients with local recurrence, as detected by I-131 WBS. For recurrent tumors that loose their iodine avidity, MRI, with very high soft tissue contrast capability, can be used to depict deep cervical and mediastinal lymph nodes since US may be insufficient due to limitations in the mediastinum and bony structures. MRI cannot replace I-131 WBS, which is the standard procedure, but it has high specificity and accuracy, especially in the mediastinum, in detecting nodal recurrence of tumors that loose their iodine avidity (26). In conclusion, it should be kept in mind that reactive changes that develop in the cervical region after thyroidectomy and RAI may hinder assessment and that the diagnostic method should be chosen according to the clinical condition of the patient. Even though the specificity of I-131 WBS is high, radioiodine positive recurrence rate is 50-60% in papillary and 64-67% in follicular thyroid cancer. In addition to MRI and cervical US, other imaging modalities such as abdominal US, bone scintigraphy, CT, and F-18 FDG PET should be employed in DTC patients with negative I-131 WBS and high serum Tg levels. F-18 FDG PET is capable of showing local recurrences as well as distant metastasis (27). Other nuclear medicine techniques that can be used to examine recurrence or metastatic focus in high-risk patients include WBS with Tl-201, Tc-99m sestamibi, and Tc-99m tetrofosmin, but the low spatial resolution associated with these techniques reduce detection of recurrence or metastasis by 25%. With a capacity of producing high-resolution images and depicting anatomic localization, F-18 FDG PET/CT stand at the forefront of these techniques. In a study on DTC patients with known or suspected recurrent disease, Middendorp and coworkers (7) examined the efficacies of F-18 FDG PET and Ga-68 DOTATOC under TSH suppression and I-131 WBS with TSH stimulation. They found that the performances of Ga-68 DOTATOC and F-18 FDG PET in radioiodine positive patients were comparable, while detection efficacy of F-18 FDG PET in radioiodine-negative patients was much higher. The authors concluded that their results needed to be supported by studies with larger series of patients and that, when recurrence is suspected, in the absence of a pathologic sign with I-131 WBS or F-18 FDG PET, Ga-68 DOTATOC PET could yield valuable information.

## CONCLUSION

We conclude that in patients with suspicion of DTC recurrence I-131 WBS is an important imaging modality that should be used, but that it can be complemented by other imaging modalities depending on the clinical condition of the patient. Even though F-18-FDG PET/CT can be used in patients with high Tg but negative I-131 WBS, we recommend obtaining I-131 WBS in conjunction with F-18 FDG PET/CT to detect recurrence when foci with or without iodine avidity co-exist.

## Ethics

Ethics Committee Approval: Ethic committee approval was not received due to a retrospective study.

Informed Consent: Consent form was filled out by all participants.

Peer-review: Externally peer-reviewed.

Financial Disclosure: The authors declared that this study has received no financial support.

## Figures and Tables

**Table 1:**
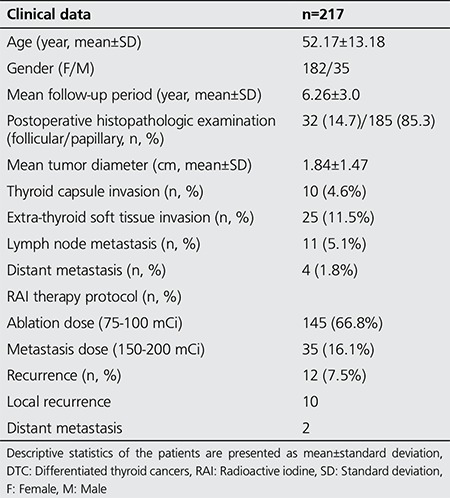
Clinical data in patients with differentiated thyroid cancers (n=217)
